# Fluid intake in urban China: results of the 2016 *Liq.In*^*7*^ national cross-sectional surveys

**DOI:** 10.1007/s00394-018-1755-5

**Published:** 2018-06-14

**Authors:** N. Zhang, C. Morin, I. Guelinckx, L. A. Moreno, S. A. Kavouras, J. Gandy, H. Martinez, J. Salas-Salvadó, G. Ma

**Affiliations:** 1 Department of Nutrition and Food Hygiene, School of Public Health, Peking University, 38 Xue Yuan Road, Haidian District, Beijing, 100191 China; 2 Laboratory of Toxicological Research and Risk Assessment for Food Safety, Peking University, Beijing, China; 3 Department of Hydration and Health, Danone Research, Palaiseau, France; 4 GENUD (Growth, Exercise, Nutrition and Development) Research Group, Faculty of Health Sciences, Universidad de Zaragoza, Zaragoza, Spain; 5 CIBERobn (Centro de Investigación Biomédica en Red Fisiopatología de la Obesidad y Nutrición), Institute of Health Carlos III, Madrid, Spain; 6 Hydration Science Lab, University of Arkansas, Fayetteville, AR USA; 7 Division of Endocrinology, University of Arkansas for Medical Sciences, Little Rock, AR USA; 8 British Dietetic Association, Birmingham, UK; 9 School of Life and Medical Services, University of Hertfordshire, Hatfield, UK; 10 Hospital Infantil de México Federico Gómez, Mexico City, Mexico; 11 Human Nutrition Unit, Biochemistry and Biotechnology Department, Hospital Universitari de Sant Joan de Reus, Faculty of Medicine and Health Sciences, IISPV (Institut d’Investigació Sanitària Pere Virgili), Universitat Rovira i Virgili, Reus, Spain

**Keywords:** Fluid types, Adequate water intake, Sugar sweetened beverages, Healthy hydration

## Abstract

**Purpose:**

To describe total fluid intake (TFI) and types of fluid consumed in urban China by age, gender, regions and city socioeconomic status relative to the adequate intakes (AI) set by the Chinese Nutrition Society.

**Methods:**

In 2016, participants aged 4–9, 10–17 and 18–55 years were recruited via a door-to-door approach in 27 cities in China. In total, 2233 participants were included. The volumes and sources of TFI were collected using the *Liq.In*^*7*^ record, assisted by a photographic booklet of standard fluid containers.

**Results:**

The mean daily TFI among children, adolescents and adults were 966, 1177 and 1387 mL, respectively. In each age group, TFI was significantly higher in male vs female (981 vs 949, 1240 vs 1113, 1442 vs 1332; mL). Approximately 45, 36 and 28% of children, adolescents and adults reached the AI. Although plain water was the highest contributor to TFI, the contribution of sugar sweetened beverages (SSB) was ranked in the top three together with water and milk and derivatives. Approximately 27, 48 and 47% of children, adolescents and adults consumed more than one serving of SSB per day, respectively.

**Conclusions:**

A relatively large proportion of participants did not drink enough to meet the AI in urban China. Many children, adolescents and adults consumed more than one serving of SSB per day. A majority of children, adolescents and adults in the study population do not meet both quantitative and qualitative fluid intake requirements, and signal socioeconomic disparities.

**Electronic supplementary material:**

The online version of this article (10.1007/s00394-018-1755-5) contains supplementary material, which is available to authorized users.

## Introduction

Water is a nutrient, which is essential for the survival and development of human’s life [1]. Water modulates normal osmotic pressure, maintains electrolyte balance and regulates body temperature. The balance between water output and water input defines hydration. Both excessive and insufficient fluid intakes have negative impacts on health. If fluid intakes exceed the capacity of renal excretion (700–1000 mL/h), it results in acute water intoxication, and probably hyponatremia. However, this is very rare and only occurs in people with psychological problems, renal disease, liver disease or congestive heart failure. Inexperienced athletes, who rapidly rehydrated after a sport event and people who drink a large amount of water to avoid heatstroke in high temperature weather are also at risk of overhydrating. By contrast, dehydration occurs when the fluid intake is insufficient to replace the free water output is more common. Even mild dehydration or a low fluid intake may impair cognitive performance [2–4], reduce the ability to perform physical activities [5, 6] and increase the incidence and prevalence of kidney and urinary system diseases [7–9]. Therefore, it is necessary to develop recommendations and guidelines for adequate intake (AI) of water and raise public recognition of the importance of maintaining an adequate water intake. The Chinese Nutrition Society has set age- and gender-specific AI for both total water intake (water from food moisture and fluids) and total fluid intake (TFI) (Table 1).

**Table 1:** Adequate total fluid intake in China (L/day) for individuals living in moderate climatic conditions with light physical activity [21]

Age (years)	Adequate intakes for total fluid intake (L/day)	
4–6	0.8	
7–10	1.0	
	Males	Females
11–13	1.3	1.1
14–17	1.4	1.2
≥ 18	1.7	1.5
Pregnant women	–	1.7
Lactating women	–	2.1

Recommendations for adequate intake of water are usually developed based, at least partly, on observed survey data. However, the importance of water is often overlooked and there are not enough studies relating to fluid intake. In China, only two surveys have been conducted that focused on TFI (the sum of drinking water and all other beverages). The first was performed among 1483 adults aged 18–60 years [10] and the second one among 5868 primary and middle school students aged 7–17 years from four cities in China [11]; the surveys were conducted in the summer of 2010. The average daily water intake of male adults (1679 mL) was higher than that of females (1370 mL). Similarly, the average daily drinking water was significantly higher in boys (1157 mL) than in girls (1026 mL). The two surveys provided vital reference data for setting the AIs. However, more fluid surveys to provide data for future revisions of the AIs in China. In both the earlier surveys, there was a significant difference in median daily TFI for adults, primary and middle school students between the cities, namely, Beijing, Shanghai, Chengdu and Guangzhou city. These findings suggest that the fluid intake of residents in different regions in China might differ. In addition, potential disparities in China by SES (socioeconomic status) need to be explored.

In addition to the amount of TFI, the type of fluid consumed by individuals is also important and needs further investigation. The sources of daily TFI usually includes tap and bottled water, milk, tea, fruit juice, sugar sweetened beverages (SSB), coffee, soft drinks and other beverages. Substantial evidence has demonstrated that different fluid types have different effects on health. Excessive SSB consumption increases risk of dental caries [12], weight gain [13], obesity [14], risk of developing the metabolic syndrome [15], type 2 diabetes [16], and dyslipidaemia [17]. Data analysis of NHANES showed that the risk of chronic kidney diseases (CKD) varied among individuals with different type of beverages consumption with lower plain water being associated with an increased risk of CKD [18, 19]. Thus, it is important to develop healthy drinking patterns based on adequate fluid intake. In China, the daily consumption of plain water, tea and other beverages has been shown to differ in adults and in children among four cities [20]. To develop further recommendations and policies on types of fluid intake to promote health drinking habits, more surveys are needed to study drinking behaviors and choices in the Chinese population.

The present study assessed the total fluid intake and type of fluids among populations of different age group in a nation-wide, urban-based sample in urban China. The primary aim of this study was to report usual daily TFI amongst the population in urban China according to age group, gender, regions and city socioeconomic status. The secondary aim was to compare TFI with adequate intakes set by the Chinese Nutrition Society [21]. And the final aim was to evaluate the contributions of different fluid types to TFI.

## Methods

### Design and study population

The present analysis reports original data collected from April 4th to May 15th 2016 by a cross-sectional survey among children (4–9 years), adolescents (10–17 years) and adults (18–55 years). This survey is part of a multinational project called *Liq.In*^*7*^ (abbreviation of *Liquid Intake over 7 days*). The primary objective of the *Liq.In*^*7*^ surveys is to assess the sources of fluid, including drinking water and different types of beverages. To ensure harmony across the surveys, standard operating procedures relating to the method of recruitment, the instruments for data collection and data treatment were developed by the co-authors and a central research private organization, and then distributed to local investigators of this private research organization. The method of recruitment, the instruments for data collection and data treatment were also harmonized with the previous *Liq.In*^*7*^ surveys [22–25]. The previous *Liq.In*^*7*^ surveys in China were performed in collaboration with the Chinese Center For Disease Control And Prevention in 2010 among adults, and in 2011 among children and adolescents [10, 11].

**Fig. 1:**
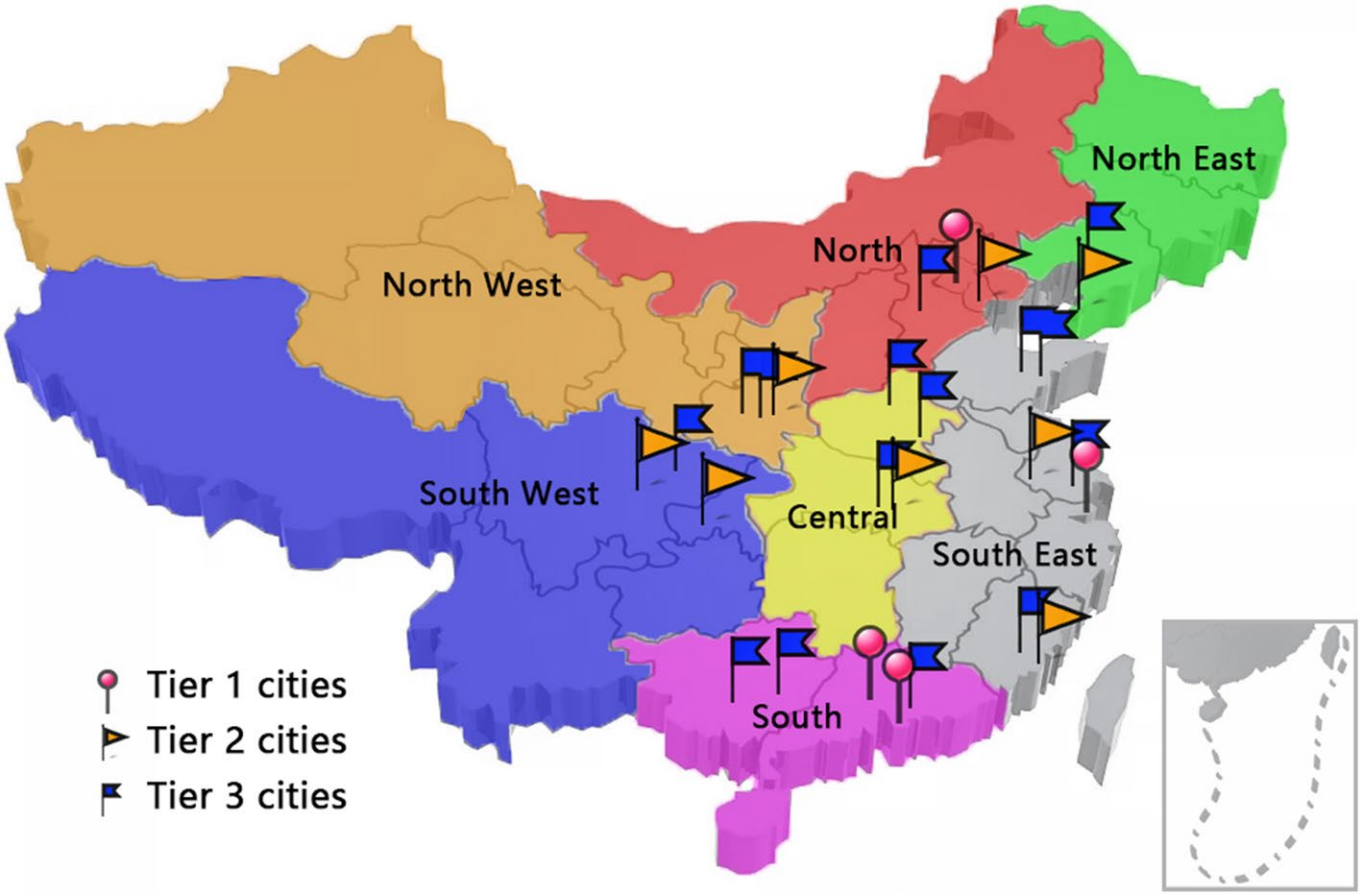
Map showing the cities in China from where participants were recruited

The participants were recruited from a database of volunteers in 27 cities via a non-systematic face-to-face and door-to-door approach until the quotas for age, gender, region of country and city socioeconomic status in relation to the total country population were met. China is classified into seven regions based on geographical location: north, northeast, east, south, northwest, southwest and central [26, 27]. The cities of different socioeconomic status were classified following the Chinese Tier City System (Fig. 1; Online Resource Table S1). This classification is based on economic development, transportation system, infrastructure and cultural significance in China according to data from National Bureau of Statistics of the People’s Republic of China [28].

Apparently healthy individuals, and only one individual per household, were eligible to participate. Individuals who were not able to read and write in the language of the questionnaire, or who were traveling within the next 10 days were not eligible to participate in the survey. Individuals working in companies advertising, marketing, market research, the media, the manufacture, distribution and/or sale of water and all kind of beverages were excluded as these individuals might be more aware of their intakes of fluids. Participants who did not complete the full 7 days of the fluid record, participants reporting a mean TFI below 0.4 L/day, participant older than 14 years reporting a TFI higher than 6 L/day and children younger than 14 years reporting a TFI higher than 4 L/day were excluded from the analysis. Pregnancy and lactation was not an exclusion criterion. Of the 8500 individuals approached, 2380 individuals who were recruited, and completed the protocol. Based on the exclusion criteria cited above, 147 individuals were excluded for analysis.

Individuals who agreed to be part of the survey received detailed information about the survey’s objectives, what was expected from them, and information about the study’s provisions to preserve confidentiality, risks and benefits, and a clear explanation about their option to participate voluntarily in the study. After being given a full description of the study, following the principles of informed consent, participants were asked for their oral approval to participate. No monetary incentive was offered for taking part in the study. All data were recorded anonymously. Therefore, participants included in the dataset cannot be identified, either directly or through identifiers. The survey protocol of the surveys was reviewed and approved by the University of Arkansas Review Board (ref. 14-12-376).

### Assessment of anthropometric variables

Height in meters (m) and weight in kilograms (kg) were measured by the researchers, and the body mass index (BMI) was calculated (kg/m^2^). BMI classification was based on BMI *z*-score for individuals aged 18 or younger [29], and the WHO classification for Asian individuals older than 19 years [30].

### Assessment of total fluid intake and the different fluid types

Participants were provided with the *Liq.In*^*7*^ record, which is a 7-day fluid-specific record validated for accuracy and reliability [31]. The *Liq.In*^*7*^ record was presented in the official country language, Chinese. The participants could choose to complete the record on paper, or via a smart phone application. The majority (95%) of participants opted to complete record via a smart phone application. Both options were explained to the participants during a face to face interview at home; they were also given written instructions. The *Liq.In*^*7*^ record was structured according to 12 moments of the day from awakening, over the different meal times and periods between meals to period during the night. The participants were instructed to report all drinking events at any moment of the day with the following details: the fluid type, the size of the container from which the fluid was drunk, the actual volume consumed, where the consumption took place and if the fluid was consumed with or without food. The record did not record consumed food. To assist the participants in estimating the actual amount of fluid consumed, a photographic booklet of standard fluid containers supported the recording. For children younger than 12 years, the primary caregiver was responsible for completing the record.

### Classification and analysis of the fluid types

The fluids recorded were classified into: water (tap and bottled water); milk and milk derivatives; hot beverages (coffee, tea and other hot beverages); 100% fruit juices; SSB; carbonated soft drinks (CSD), juice-based drinks, functional beverages such as energy and sports drinks, ready to drink tea and coffee and flavored water; artificial/non-nutritive sweeteners beverages (A/NSB) (diet/zero soft drinks); and other beverages. A more detailed classification can be found in Online Resources Table S2 of this paper. TFI was defined as the sum of all these categories.

The proportion of individuals drinking less or more than adequate intake of total fluid intake set by the Chinese Nutrition Society was calculated (Table 1). To allow comparison with previously published data, the comparison between observed intakes and the adequate intake of water from fluids set by the European Food Safety Authority is also provided in the Online Resources Fig S1 [32].

The proportion of individuals drinking ≤ 1 serving (being 250 mL) of SSB per week, 2–6 servings of SSB per week and ≥ 1 serving of SSB per day was calculated. These cut-offs were determined from meta-analyses associating such intakes with potential risks for the development of obesity, type 2 diabetes and metabolic syndrome [33–35]. An individual was considered a consumer of a certain fluid types if the individual reports the consumption of fluid types at least once during 7 days.

### Statistical analysis

The demographic and anthropometrics characteristics of the study population are presented either as means and standard deviation for continuous variables, or numbers and percentages for dichotomous variables. As all intakes are skewed data (Online Resource Fig. S2), TFIs are presented both as mean and standard error of mean, as well as all percentiles of intake. The intake of different fluids are presented as median (50th percentile) and percentile 25th and 75th (Table 4 and Online Resource Table S3). The contribution (%) of each fluid type to TFI was calculated as 7-day mean intake of that fluid type divided by the 7-day mean of TFI. The mean and standard error of mean, as well as the contribution of the different fluid types to TFI according to age groups and gender can be found as Online Resources Tables S4 and S5. Intakes are estimated values of all participants, including non-consumers. The data were simply combined, without application of weights. Comparisons of TFI by gender, region and city socioeconomic status were made with a Wilcoxon rank test. All statistical tests were two-tailed and the significance level was set at *P* < 0.05. The analyses were performed using the SPSS software version 22.0 (SPSS Inc., Chicago, IL, USA) and were verified by a statistician.

## Results

### Sample description

The baseline demographic and anthropometrics characteristics of participants are summarized in Table 2. A total of 2233 participants (1120 males and 1113 females) aged from 4 to 55 years old in urban China were included. The proportions of different age groups (children, adolescents and adults) were 12, 17 and 71%, respectively, with the similar male to female ratios (1:1, approximately). Almost one-fourth of the children and adults were overweight; the highest percentage of obesity (18%) was observed among the children. The distributions of participants in the three age groups were similar in the different regions (*p* = 0.98), with the proportion of children in each region ranging from 5 to 25%, adolescents 6–25%, and adults 6–25%. The distributions of participants in the three age groups in cities with different socioeconomic status differed significantly (*p* = 0.05).

**Table 2:** Demographic and anthropometric characteristics of the survey population (*N* = 2233), by age

	4–9 years	10–17 years	18–55 years
Sample size^a^	279 (12%)	370 (17%)	1584 (71%)
Males	143 (51%)	187 (51%)	790 (50%)
Females	136 (49%)	183 (49%)	794 (50%)
Age^b^ (years)	6.3 ± 1.6	13.7 (2.4)	33.7 (10.2)
BMI^b^ (kg/m^2^)	19.4 ± 6.3	21.2 (5.9)	23.2 (5.6)
BMI classification^a,c^			
Underweight	8 (3%)	10 (3%)	98 (6%)
Normal	102 (37%)	218 (59%)	708 (45%)
Overweight	65 (23%)	63 (17%)	380 (24%)
Obesity	50 (18%)	20 (5%)	105 (7%)
No data	54 (19%)	59 (16%)	293 (18%)
Region^a^			
North	38 (14%)	52 (14%)	208 (13%)
Northeast	13 (5%)	22 (6%)	99 (6%)
East	70 (25%)	94 (25%)	389 (25%)
South	46 (16%)	53 (14%)	235 (15%)
Northwest	35 (13%)	39 (11%)	180 (11%)
Southwest	43 (15%)	71 (19%)	286 (18%)
Central	34 (12%)	39 (11%)	187 (12%)
City socioeconomic status^a,d^			
Tier 1	65 (23%)	99 (27%)	395 (25%)
Tier 2	89 (32%)	150 (41%)	607 (38%)
Tier 3	125 (45%)	121 (33%)	582 (37%)

*SD* standard deviation, *BMI* body mass index

^a^Data are expressed as numbers (percentage) for categorical variables

^b^Data are presented as mean ± SD for continuous variables

^c^BMI classification is based on BMI *z*-score for individuals aged 4–18 years and the Asian classification for individuals older than 19 years

^d^Classification of city socioeconomic status is based on Chinese Tier city system, which take into account of the aspects of economic development, transportation system, infrastructure and cultural significance in China and refer to the data of National Bureau of Statistics of the People’s Republic of China

### Daily total fluid intake

The mean daily TFI for children, adolescents and adults were 966 mL (839 [611–1196]), 1177 mL (1071 [775–1408]) and 1387 mL (1214 [889–1684]), respectively (Table 3). Among children and adolescents, no significant differences were observed for daily TFI between genders. However, for adults, males consumed significantly more than females (1442 vs 1332 mL/day, *p* = 0.004). A significant difference in TFI was found between different regions (*p* < 0.0001), as well as between the cities with different socioeconomic status (*p* < 0.0001).

**Table 3:** Daily total fluid intake (mL/day) among children (4–9 years), adolescents (10–17 years) and adults (18–55 years) by gender, region and city socioeconomic status

Target	Gender	*N* (%)	Mean TFI ± SEM	Percentiles							*p* value^a^
				5	10	25	50	75	90	95	
Age group (years)											
4–9	Total	279	966 ± 30	420	497	611	839	1196	1648	1874	*NS*
	Males	143 (51%)	981 ± 38	420	459	614	891	1286	1635	1808	
	Females	136 (49%)	949 ± 47	417	509	607	784	1105	1702	1999	
10–17	Total	370	1177 ± 31	481	579	775	1071	1408	1885	2373	*NS*
	Males	187 (51%)	1240 ± 46	497	584	777	1111	1550	2147	2624	
	Females	183 (49%)	1113 ± 42	475	554	774	986	1328	1674	2042	
18–55	Total	1584	1387 ± 18	567	678	889	1214	1684	2323	2794	*NS*
	Males	790 (50%)	1442 ± 28	567	702	908	1263	1760	2450	2952	
	Females	794 (50%)	1332 ± 24	566	662	870	1163	1624	2219	2633	
Region											
North		298 (13%)	1431 ± 40	608	724	945	1261	1767	2315	2700	*p* < 0.0001
Northeast		134 (6%)	1429 ± 58	586	708	934	1302	1801	2217	2618	
East		553 (25%)	1239 ± 29	520	613	778	1064	1504	2071	2573	
South		334 (15%)	1540 ± 42	612	752	999	1357	1907	2613	3147	
Northwest		254 (11%)	1231 ± 38	507	582	792	1082	1544	1957	2333	
Southwest		400 (18%)	1121 ± 30	438	537	719	1000	1331	1815	2364	
Central		260 (12%)	1246 ± 47	463	552	773	1052	1449	2284	2886	
City socioeconomic status^b^											
Tier 1		559 (25%)	1483 ± 30	613	724	975	1328	1833	2415	2904	*p* < 0.0001
Tier 2		846 (38%)	1233 ± 22	534	613	793	1075	1488	1956	2485	
Tier 3		828 (37%)	1244 ± 25	470	558	765	1083	1496	2134	2686	

*NS n*ot statistically significant, *SEM* standard error of the mean, *SES* socioeconomic status

^a^Wilcoxon test to compare means among regions or cities with different socioeconomic

^b^Classification of city socioeconomic status is based on Chinese Tier city system, which takes into account the aspects of economic development, transportation system, infrastructure and cultural significance in China and refers to the data of National Bureau of Statistics of the People’s Republic of China. Tier 1 is the highest (wealthiest) SES group

### Comparison with adequate intake of water from fluids set by the Chinese Nutrition Society

**Fig. 2:**
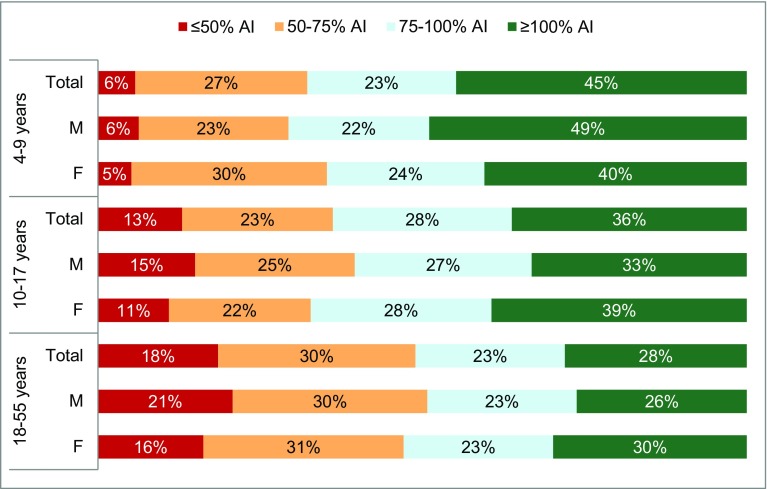
Percentage (%) of participants according to adherence categories of adequate intake of water from fluids set by the Chinese Nutrition Society among children (4–9 years), adolescents (10–17 years) and adults (18–55 years). *M* Males, *F* Females

Figure 2 shows the proportion of participants in the three age groups according to gender, consuming > 100, 75–100, 50–75 and ≤ 50% of the AI of water from fluids set by Chinese Nutrition Society. The percentage of meeting the AIs was highest in children (45%), with 36% for adolescents and 28% for adults. A higher proportion of males achieved the adequate intake than female for adolescents and adults (39 vs 33%; 30 vs 26%; respectively). More females achieved an adequate intake than males (49 vs 40%).

A higher proportion of participants living in the Tier 1 adhere to the adequate intake than participants in Tier 2 and 3 (44 vs 27 and 28%, respectively) (Online resources Figs. S3, S4).

The, the northeast and the south regions had the highest proportion of people achieving the adequate intake (41, 38 and 47%; respectively) and the southwest and the central regions had the lowest (21 and 24%; respectively) (Online resources Fig S5).

### Daily intake of different fluid types

**Fig. 3:**
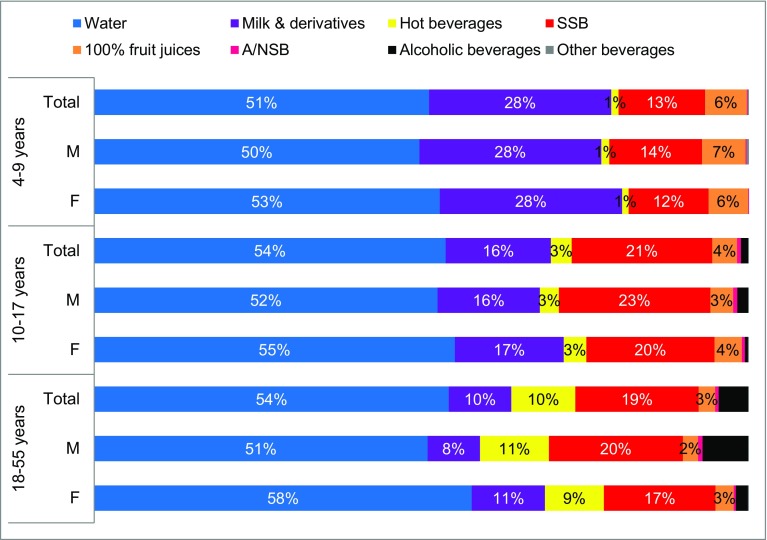
Contribution of the different fluid types to TFI (%) among children (4–9 years), adolescents (10–17 years) and adults (18–55 years)

The median intake and the contribution of the different fluid types to TFI are shown in Table 4 and Fig. 3. The main contributor of TFI was plain water (approximately 50% of TFI), either bottled or tap, with a median intakes ranged 403 (245–658) mL/day for children and 597 (349–961) mL/day for adults. In all age groups water intake represented above half of TFI. The second highest contributor to TFI was milk and derivatives among children and SSBs among adolescents and adults (Fig. 3).

**Table 4:** Median daily intake (mL/day) of different fluid types of males and females among children (4–9 years), adolescents (10–17 years) and adults (18–55 years)

	4–9 years				10–17 years				18–55 years			
	Males		Females		Males		Females		Males		Females	
	Median (P25–P75)	% consumers	Median (P25–P75)	% consumers	Median (P25–P75)	% consumers	Median (P25–P75)	% consumers	Median (P25–P75)	% consumers	Median (P25–P75)	% consumers
Water	400 (229–666)	98%	403 (266–620)	99%	540 (324–789)	100%	477 (284–763)	100%	569 (337–922)	100%	632 (360–997)	99%
Bottled water	48 (0–186)	67%	35 (0–155)	57%	143 (0–357)	73%	104 (17–329)	77%	149 (36–374)	81%	122 (8–349)	75%
Tap water	240 (96–518)	90%	303 (170–463)	93%	271 (81–516)	92%	256 (105–509)	90%	284 (71–586)	88%	363 (120–687)	90%
Milk and derivatives	239(153–388)	95%	223 (126–334)	96%	170 (64–271)	87%	157 (70–286)	91%	78 (3–171)	75%	131 (50–218)	85%
Hot beverages	0 (0–0)	19%	0 (0–0)	15%	0 (0–36)	36%	0 (0–42)	35%	36 (0–150)	62%	36 (0–119)	60%
Coffee	0 (0–0)	6%	0 (0–0)	4%	0 (0–0)	16%	0 (0–0)	14%	0 (0–27)	31%	0 (0–34)	34%
Tea	0 (0–0)	15%	0 (0–0)	11%	0 (0–26)	28%	0 (0–29)	27%	0 (0–108)	50%	0 (0–71)	43%
SSB	91 (0–207)	72%	68 (0–140)	71%	222 (69–377)	86%	175 (46–333)	84%	229 (73–430)	85%	163 (51–328)	83%
CSD	0 (0–34)	31%	0 (0–0)	23%	0 (0–104)	50%	0 (0–64)	40%	0 (0–86)	47%	0 (0–50)	39%
Juice–based drinks	0 (0–63)	48%	0 (0–62)	48%	0 (0–70)	47%	26 (0–79)	56%	0 (0–64)	40%	0 (0–69)	46%
Functional beverages	7 (0–82)	50%	0 (0–47)	44%	36 (0–147)	58%	36 (0–116)	56%	36 (0–129)	57%	0 (0–71)	46%
RTD tea and coffee	0 (0–18)	28%	0 (0–0)	21%	0 (0–79)	47%	11 (0–71)	51%	41 (0–*12*9)	59%	36 (0–107)	62%
Flavored water	0 (0–0)	3%	0 (0–0)	1%	0 (0–0)	5%	0 (0–0)	6%	0 (0–0)	4%	0 (0–0)	4%
100% fruit juices	38 (0–93)	62%	8 (0–73)	52%	0 (0–60)	41%	7 (0–80)	51%	0 (0–42)	37%	0 (0–54)	41%
A/NSD	0 (0–0)	5%	0 (0–0)	2%	0 (0–0)	13%	0 (0–0)	7%	0 (0–0)	10%	0 (0–0)	7%
Alcoholic beverages	0 (0–0)	0%	0 (0–0)	0%	0 (0–0)	11%	0 (0–0)	8%	0 (0–107)	45%	0 (0–0)	21%
Other beverages	0 (0–0)	4%	0 (0–0)	1%	0 (0–0)	2%	0 (0–0)	3%	0 (0–0)	3%	0 (0–0)	4%

*SSB* sugar sweetened beverages, *CSD* carbonated sweetened beverages, *RTD* ready to drink, *A*/*NSD* artificial/non-nutritive sweetened beverages, *P25* the 25th percentile, *P75* the 75th percentile

Among children and adolescents, there were no statistical differences in daily intakes of various fluid types between males and females (Online resources Table S2). Amongst adults, the mean daily intake of SSB was significantly higher in males [295 (11) mL/day] compared with females [227 (9) mL/day; *p* < 0.0001]. The mean daily intakes of water (*p* = 0.01) and milk and derivatives (*p* < 0.0001) were significantly higher in female adults than male adults. The fluid types with the highest percentage of consumers were, in order of volume, water, milk and derivatives, SSB and hot beverages.

**Fig. 4:**
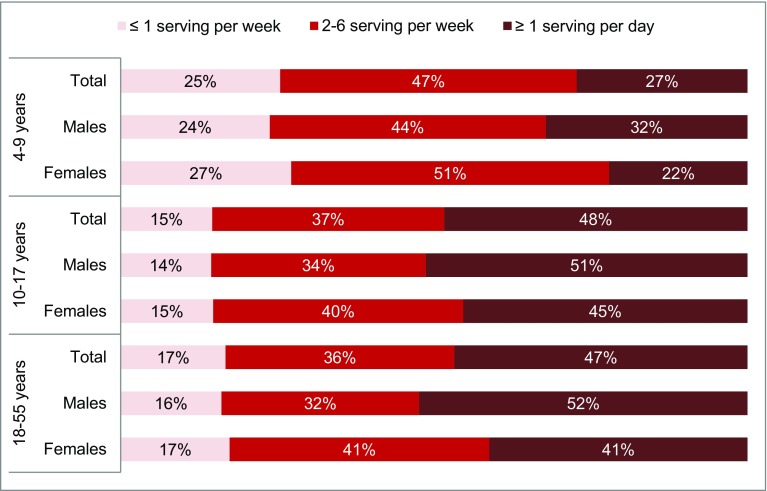
Percentage of children (4–9 years), adolescents (10–17 years) and adults (18–55 years) drinking 1 serving (250 mL) of SSB daily or less per week, 2–6 servings per week and 1 serving or more per day, by gender

In addition, on average 48% of adolescents and 47% of adults consumed ≥ 1 servings of SSB/day while a consumption of ≥ 1 servings of SSB/day was observed for only 27% of children (Fig. 4). Almost half of the children (47%) drank 2–6 servings of SSB/week, while 37 and 36% of adolescents and adults drank this amount. One-fourth of the children, 15% of adolescents and 17% of adults drank ≤ 1 serving of SSB/week. Among children, adolescents and adults, there are, respectively, 28, 15 and 16% of individuals reporting zero SSB intake.

## Discussion

The main objectives of the present study were to estimate the 7-day mean TFI and the different fluid types among a relatively large sample across seven regions based on geographical location in urban China according to age group, gender, regions and cities with different socioeconomic status, to assess the percentage of participants who comply with the adequate intake of water from fluids set by Chinese Nutrition Society, and to evaluate the contributions of fluid types to TFI.

Less than a half of children, approximately a third of adolescents and merely about a quarter of adult participants in this study reached the amount of AI, which means that a considerable portion of participants might be at the risk of being under-hydrated. In the previous fluid intake survey with the *Liq.In*^*7*^ record of children and adolescent in four Chinese cities conducted in China, the TFI of children and adolescents aged 7–17 years was 1086 mL/day which is higher than the TFI of children (966 mL/day) [36] and lower than TFI of adolescents (1177 mL/day) in the current study [10]. For adults, the TFI in current survey differed by only approximately 100 mL/day compared with the fluid intake survey of adults in four Chinese cities (1584 vs 1488, mL/day) in 2010 [20]. In a survey with the *Liq.In*^*7*^ record specifically conducted in young adults (aged 18–25 years) in China in 2015, TFI was 1342 mL/day and less than the adults in this survey [37]. In a systematic review including 273 studies in different countries (only one Asia survey in China was included; the majority being USA and European surveys), it was concluded that TFI varied between 0.6 and 3.5 mL/day in the general population and 0.6–1.8 mL/day amongst children, 0.8–2.0 mL/day amongst adolescents, and 0.8–3.4 mL/day amongst adults [38]. Another survey using the *Liq.In*^*7*^ record in 2015 also indicated that only approximately a quarter of the Chinese young adults met the recommendation [37]. Harmonized cross-sectional fluid specific surveys performed in 15 countries showed that a relatively high proportion of subjects (38%) failed to meet the recommendation on fluid intake and this proportion significantly varied among countries [39]. However, the recommendation used in harmonized surveys was the EFSA AIs, not Chinese AI as used in the recent study; this may partly explain the different adherence levels were. The present survey suggests that some people in China are not meeting the AI although the exact prevalence requires further study.

In the present study, water was the fluid type consumed in the highest volume, followed by SSB and milk and its derivatives. In the fluid intake survey with the *Liq.In*^*7*^ record of children and adolescent in four Chinese cities in 2010, the contribution of water to TFI was also the highest in primary and middle school students and adults [36] (68.3 and 60.3%). It is important to note that 27, 48 and 47% of children, adolescents and adults consumed more than one serving SSB per day, respectively, an intake, suggested by some studies that may result in harmful effects on health such as weight gain and dental caries [40, 41]. Unsweetened tea is a suitable alternative to water and favored by residents in China; tea contributed nearly ten percent of TFI in this survey. In a systematic review including 273 studies, water contributed up to 58, 75 and 80% of TFI among children, adolescents and adults [38]. In a fluid specific survey with the *Liq.In*^*7*^ record including 16,276 adults across 13 countries from three continents, the fluid with the highest intake was also water (except in Argentina, UK, Poland and Japan) and water intake ranged from 270 mL/day in Japan to 1780 mL/day in Indonesia; the second and third mostly consumed fluid was hot beverages and regular sweetened beverages in most countries [19]. In the fluid intake survey of children and adolescent in four Chinese cities in 2010, the consumption of water in high school students was the highest (829 mL/day) and that in primary students was the lowest (672 mL/day) [36]. In the aforementioned systematic review, TFI increased gradually among children, adolescents and adults with age; the consumption of fluid types varied according to age, whilst children consumed more milk, adolescents consumed more soft drinks, and adults drank more tea, coffee and alcoholic beverages [38]. In the harmonized cross-sectional surveys of fluid intake using the *Liq.In*^*7*^ record, it was shown that children and adolescents were less likely to meet the recommendation of fluid intake than adults and the contribution of juices and sweet beverages was as high as the contribution of water to TFI [39].

In this survey, it was shown that children and male adolescents chose healthier fluid types (more water and less SSB) than adolescent girls, while amongst adults women preferred better fluid types than men. Gender differences in TFI have previously been shown in primary and middle school students and adults in previous two surveys in China [36] with adult men drinking more tea than women.

Comparisons between regions showed that there were significant differences on TFI, and TFI was highest in the south region of China. In terms of city different socioeconomic status, differences in TFI were also found among cities with different socioeconomic status with TFI being the highest in Tier 1 cities. The data might also suggest that lower SES groups (Tier 2 cities and Tier 3 cities) are disproportionately less likely to meet the AI. This is an important and novel finding as previous studies have not compared the even regions nor cities with different socioeconomic status in China. The landscapes and geographical location vary significantly across the vast width of China, which may result in the differences in TFI due to the different altitudes and environmental conditions. Analyzing the data from different regions may provide useful detailed data for future revisions of the water AI in China according to the geographical location. In the fluid intake survey with the *Liq.In*^*7*^ record of adults in four Chinese cities in 2010, TFI was highest in Shanghai (east, 1793 mL/day), while least in Chengdu (west, 1150 mL/day); water intake was highest in Guangzhou (south, 917 mL/day); tea intake was highest in Shanghai; beverage consumption (including all types of beverages except water and tea) was higher in Shanghai and Beijing (north) (323 and 264, mL/day) [42]. In the survey across 13 countries, there were considerable differences in the consumption of the different fluid types among countries; however, relatively similar fluid intake patterns were observed among countries located in the neighboring geographical area. It is important to recognize there may be discrepancies of educational level and socioeconomic status among cities with different socioeconomic status defined by the Chinese government based on the combination of GDP, administrative authorities and other economic criteria such as population size. In the previous fluid intake survey using the *Liq.In*^*7*^ record of adults in four Chinese cities in 2010, water intake in urban areas was less than rural ones, which was opposite for tea and beverages. In addition, educational level had an effect on the volume of beverages consumed [42]. Some surveys have analyzed the effect of educational level and socio-economics status on TFI, and found that socioeconomic status was associated with TFI. In a study among US adults, it was observed that lower income adults had a higher risk of inadequate hydration than higher income adults [43].

There were some strengths and limitations in the present study. In terms of strengths, this analysis was unique as it collected data on TFI of a relatively large sample with an equal gender distribution covering a relatively large part of China. This provides more representative data for developing recommendations and guidelines of adequate water intake. In addition, the study explores potential disparities among various populations. The use of a photographic booklet of standard fluid containers limited the self-reporting bias. The *Liq.In*^*7*^ used in this survey has been validated for accuracy and reliability; however, this validation was performed in an American, adult population [31]. Several limitations of the survey or the analysis need to be acknowledged. There is the potential for sampling design limitations, such as bias that may result if individuals who are not at home share characteristics that differ from those who are at home. The disadvantage of using a harmonized protocol is that some methods were used to enable inter-country comparisons that might not have been optimally sensitive for use in China; for example, the analysis protocol group beverages using the same categories as used in other countries, not China-specific categories. The prevalence of overweight and obese in the children sample was higher than that observed in the Chinese National Survey on Students Constitution and Health in 2014 [44], and the proportion of participants living in urban areas was higher in this survey. Rural population was not included in this survey. Thus, the results may not be generalizable to whole population on a nationwide scale. Fluid intake from foods and food data were not assessed in the study, which prevents the estimation of total water intake. However, it might have increased the quality of the fluid data reported, as participants were focused on reporting all drinking acts. If participants had to report all food and fluids intake, drinking acts outside the regular meals may have been missed [45]. Physical activities of subjects have not been investigated. Weighted analysis has not used in this study.

In conclusion, the current survey showed that TFI varies by gender, region and city socioeconomic status in urban China. A relatively large proportion of participants in the survey drank less than the AI of water from fluids, and are, therefore, potentially at risk of under-hydration. About half of the adolescents and adults consumed ≥ 1 serving SSB/day, an intake that may be associated with adverse effects on health. The results have potential implications for health disparities and emphasize the need for interventions that target particular groups, such as lower SES groups or particular regions. Effective interventions should be developed and implemented to increase fluid intake and improve beverage choice, while analyzing their efficacy. Health promotion policies and campaigns should encourage the creation of an environment favoring water consumption.

## Electronic supplementary material

Below is the link to the electronic supplementary material.

Supplementary material 1 (DOCX 61 KB)
