# Supplementary material for: Fluid intake in urban China: results of the 2016 Liq.In7 national cross-sectional surveys
Source: Eur J Nutr. 2018 Jun 14;57(Suppl 3):77–88. doi: 10.1007/s00394-018-1755-5 (PMC6008349; doi:10.1007/s00394-018-1755-5)
Supplement: Supplementary file 1 — Supplementary material 1 (DOCX 61 KB) [file 394_2018_1755_MOESM1_ESM.docx]

**FLUID INTAKE IN URBAN CHINA: RESULTS OF THE 2016 LIQ.IN^7^ NATIONAL CROSS-SECTIONAL SURVEYS**

*Zhang N,^1,2^ Morin C,^3^ Guelinckx I, ^3^ Moreno LA,^4,5^ Kavouras SA,^6,7^ Gandy J,^8,9^ Martinez H,^10^ Salas-Salvadó J,^5,11^ Ma G, ^1^*^,2*^

^1^ Department of Nutrition and Food Hygiene, School of Public Health, Peking University, Beijing, China

^2^ Laboratory of Toxicological Research and Risk Assessment for Food Safety, Peking University, Beijing, China

^3^ Department of Hydration & Health, Danone Research, Palaiseau, France

^4^ GENUD (Growth, Exercise, Nutrition and Development) Research Group, Faculty of Health Sciences, Universidad de Zaragoza, Zaragoza, Spain

^5^ CIBERobn (Centro de Investigación Biomédica en Red Fisiopatología de la Obesidad y Nutrición), Institute of Health Carlos III, Madrid, Spain

^6^ Hydration Science Lab, University of Arkansas, Fayetteville, AR

^7^ Division of Endocrinology, University of Arkansas for Medical Sciences, Little Rock, AR

^8^ British Dietetic Association, Birmingham, UK

^9^ School of Life and Medical services, University of Hertfordshire, Hatfield, UK

^10^ Hospital Infantil de México Federico Gómez, México City, México

^11^ Human Nutrition Unit, Hospital Universitari de Sant Joan de Reus, Faculty of Medicine and Health Sciences, IISPV (Institut d’Investigació Sanitària Pere Virgili), Biochemistry and Biotechnology Department, Universitat Rovira i Virgili, Reus, Spain

*** Corresponding author:** Prof Ma G, Department of Nutrition and Food Hygiene, School of Public Health, Peking University, Beijing, China; 38 Xue Yuan Road, Haidian District, Beijing 100191, China; mags@bjmu.edu.cn; Tel.: +86-10-8280-5266

**ONLINE RESOURCES**

**Table S1** Classification of cities according to Chinese Tier City System, proxy for demographic and social segmentation

| Tier 1 | Tier 2 | Tier 3 |
| --- | --- | --- |
| Beijing | Changle-Fuzhou | Anxi |
| Guangzhou | Chengdu | Baoji |
| Shanghai | Chongqing | Dingzhou |
| Shenzhen | Dalian | Gaomi |
|  | Nanjing | Guigang |
|  | Nantong | Hanchuan |
|  | Tianjin | Huaiyang |
|  | Wuhan | Jiangyou |
|  | Xiamen | Liaoyang |
|  | Xi'an | Luoding |
|  |  | Wuming |
|  |  | Xingping |
|  |  | Xinxiang |

**Table S2** Classification of the fluid types

| Classification | Detailed Fluid types |
| --- | --- |
| **Water** |  |
| *Bottled water* | Unflavored still water, unflavored sparkling water |
| *Tap water* | Tap water, filtered water, boiled water |
| **Milk & derivatives** | Low fat and full fat milk, fermented milk, ready-to-drink milk, flavored milk, yogurt milk, raw milk, powder milk, powder/syrup flavored milk, fruit shake with milk, cocoa compound with milk, almond/rice milk |
| **Hot beverages** |  |
| *Coffee* | Coffee, coffee from coffee maker (e.g.: homemade coffee, dolce gusto, others), powder coffee, instant coffee, vending machine coffee, restaurant/franchise coffee, |
| *Tea* | Homemade hot/cold tea (from tea bags), infusions (herbal) |
| **SSB** |  |
| *Carbonated sweet beverages* (*CSD)* | Cola carbonated drinks, tonic, soda |
| *Juice-based drinks* | Packages fruits & vegetables juices (packages juices (fruits or vegetables), packaged orangeade, packages nectars, Eskimos/smoothies), flavored carbonated drinks flavored sparkling water, fruit shake with water powder, flavored waters made with powder or concentrate/syrup, |
| *Functional beverages* | Sports drinks (such as Aquarius, etc.), vitamin/ functional drinks (fiber, vitamin and cooling drinks such as C1000 Vitamin Lemon), energy drinks, protein drinks, liquid or powder isotonics |
| *RTD tea & coffee* | Bottled coffee, ready to drink bottled tea, ready to drink ice tea, bubble tea |
| *Flavored water* | Flavored packaged water, cocoa compound with water, Infused / herbal / vegetal water |
| **100% fruit juices** | Bottled 100% fruit juice & vegetables juice, homemade freshly squeezed juice, Freshly squeezed juice "take from outside", |
| **A/NSB** | Diet sweet beverages (packaged light juices, diet/light/zero cola carbonated drinks, diet/light/zero flavored carbonated drinks), beverages sweetened with stevia or other non-nutritive sweeteners, packaged light fruit juice/nectar/vegetable, |
| Alcoholic beverages | Beer, malt beer, beer mix drinks, wine, champagne, aperitifs and digestives straight, packages/canned alcoholic beverages, alcoholic beverages straight (not mixed like vodka, tequila, rum, ….), aperitifs and digestives with other beverages |
| **Other beverages** | - Beverages identified by participant as “other than listed above” - Packaged soy drinks, diet drinks as meal replacement(slim fast), ready to drink soy based juice, beer 0% alcohol |

**Table S3** Median daily intake (mL/day) of different fluid types and the percentage of consumers among children (4-9 years), adolescents (10-17 years) and adults (18-55 years)

|  | **4-9 years** | | **10-17 years** | | **18-55 years** | |
| --- | --- | --- | --- | --- | --- | --- |
|  | Median  (P25-P75) | % consumers | Median  (P25-P75) | % consumers | Median  (P25-P75) | % consumers |
| **Water** | 403 (245-658) | 98% | 518 (303-777) | 100% | 597 (349-961) | 99% |
| *Bottled water* | 43 (0-161) | 62% | 131 (6-344) | 75% | 137 (21-363) | 78% |
| *Tap water* | 286 (113-489) | 91% | 262 (98-513) | 91% | 321 (96-640) | 89% |
| **Milk & derivatives** | 227 (136-356) | 96% | 161 (68-278) | 89% | 107 (32-200) | 80% |
| **Hot beverages** | 0 (0-0) | 17% | 0 (0-36) | 36% | 36 (0-129) | 61% |
| *Coffee* | 0 (0-0) | 5% | 0 (0-0) | 15% | 0 (0-32) | 33% |
| *Tea* | 0 (0-0) | 13% | 0 (0-26) | 28% | 0 (0-91) | 46% |
| **SSB** | 71 (0-179) | 71% | 205 (64-363) | 85% | 193 (68-385) | 84% |
| *CSD* | 0 (0-16) | 27% | 0 (0-79) | 45% | 0 (0-71) | 43% |
| *Juice-based drinks* | 0 (0-63) | 48% | 8 (0-71) | 52% | 0 (0-64) | 43% |
| *Functional beverages* | 0 (0-71) | 47% | 36 (0-126) | 57% | 18 (0-107) | 51% |
| *RTD tea & coffee* | 0 (0-0) | 25% | 0 (0-76) | 49% | 36 (0-116) | 61% |
| *Flavored water* | 0 (0-0) | 3% | 0 (0-0) | 6% | 0 (0-0) | 4% |
| **100% fruit juices** | 21 (0-81) | 57% | 0 (0-71) | 46% | 0 (0-50) | 39% |
| **A/NSD** | 0 (0-0) | 4% | 0 (0-0) | 10% | 0 (0-0) | 9% |
| **Alcoholic beverages** | 0 (0-0) | 0% | 0 (0-0) | 10% | 0 (0-43) | 33% |
| **Other beverages** | 0 (0-0) | 3% | 0 (0-0) | 2% | 0 (0-0) | 3% |

*SSB* sugar sweetened beverages, *CSD* carbonated sweetened drinks, *RTD* ready to drink, *A/NSB* artificial/non-nutritive sweetened beverages

**Table S4** Mean daily intake (SEM) of different fluid types (mL/day) among children (4-9 years), adolescents (10-17 years) and adults (18-55 years) by gender

|  | 4-9 years | | | | | | 10-17 years | | | | | | 18-55 years | | | | | |
| --- | --- | --- | --- | --- | --- | --- | --- | --- | --- | --- | --- | --- | --- | --- | --- | --- | --- | --- |
|  | Total | | Males | | Females | | Total | | Males | | Females | | Total | | Males | | Females | |
| **TFI** | **966** | **±30** | **981** | **±38** | **949** | **±47** | **1177** | **±31** | **1240** | **±46** | **1113** | **±42** | **1387** | **±18** | **1442** | **±28** | **1332** | **±24** |
| Water | 494 | ±21 | 487 | ±29 | 501 | ±31 | 632 | ±27 | 650 | ±37 | 613 | ±40 | 751 | ±16 | 734 | ±23 | 768 | ±21 |
| *Bottled water* | *129* | *±13* | *130* | *±16* | *127* | *±20* | *229* | *±16* | *230* | *±21* | *227* | *±23* | *270* | *±9* | *282* | *±14* | *258* | *±13* |
| *Tap water* | *365* | *±20* | *357* | *±30* | *374* | *±27* | *403* | *±27* | *420* | *±38* | *386* | *±38* | *482* | *±15* | *453* | *±22* | *510* | *±20* |
| Milk & derivatives | 269 | ±12 | 273 | ±16 | 265 | ±19 | 189 | ±8 | 194 | ±12 | 185 | ±11 | 132 | ±3 | 116 | ±4 | 149 | ±5 |
| Hot beverages | 11 | ±2 | 12 | ±3 | 9 | ±4 | 37 | ±6 | 36 | ±7 | 38 | ±9 | 136 | ±7 | 152 | ±11 | 120 | ±8 |
| *Coffee* | *2* | *±1* | *2* | *±1* | *1* | *±1* | *9* | *±2* | *9* | *±2* | *10* | *±2* | *24* | *±1* | *22* | *±2* | *25* | *±2* |
| *Tea* | *9* | *±2* | *10* | *±3* | *8* | *±4* | *28* | *±5* | *27* | *±6* | *29* | *±9* | *112* | *±7* | *130* | *±11* | *94* | *±8* |
| SSB | 128 | ±12 | 139 | ±14 | 116 | ±20 | 253 | ±13 | 287 | ±21 | 218 | ±14 | 261 | ±7 | 295 | ±11 | 227 | ±9 |
| *CSD* | *19* | *±3* | *25* | *±5* | *13* | *±3* | *58* | *±5* | *71* | *±8* | *44* | *±6* | *53* | *±3* | *67* | *±4* | *39* | *±3* |
| *Juice-based drinks* | *45* | *±5* | *43* | *±6* | *47* | *±8* | *50* | *±4* | *50* | *±6* | *50* | *±5* | *43* | *±2* | *41* | *±3* | *45* | *±3* |
| *Functional beverages* | *47* | *±5* | *50* | *±6* | *43* | *±8* | *85* | *±6* | *101* | *±11* | *69* | *±7* | *73* | *±3* | *92* | *±5* | *55* | *±3* |
| *RTD tea & coffee* | *16* | *±3* | *19* | *±4* | *13* | *±5* | *54* | *±4* | *58* | *±7* | *51* | *±6* | *89* | *±4* | *92* | *±5* | *86* | *±5* |
| *Flavored water* | *1* | *±0* | *1* | *±1* | *1* | *±1* | *5* | *±2* | *7* | *±4* | *3* | *±1* | *2* | *±0* | *2* | *±0* | *3* | *±1* |
| 100% fruit juices | 61 | ±7 | 65 | ±8 | 57 | ±10 | 45 | ±4 | 43 | ±6 | 46 | ±5 | 35 | ±2 | 34 | ±2 | 37 | ±2 |
| A/NSD | 1 | ±0 | 2 | ±1 | 1 | ±1 | 7 | ±1 | 8 | ±2 | 5 | ±2 | 7 | ±1 | 10 | ±3 | 4 | ±1 |
| Alcoholic beverages | 0 | ±0 | 0 | ±0 | 0 | ±0 | 13 | ±4 | 21 | ±7 | 5 | ±2 | 62 | ±4 | 100 | ±7 | 25 | ±3 |
| Other beverages | 2 | ±1 | 3 | ±2 | 0 | ±0 | 1 | ±1 | 1 | ±1 | 2 | ±1 | 2 | ±0 | 2 | ±0 | 2 | ±0 |

*SSB* Sugar sweetened beverages, *CSD* Carbonated sweetened beverages, *RTD* Ready to drink, *A/NSD* Artificial/non-nutritive sweetened beverages

**Table S5** Contribution (%) of different fluid types to Total Fluid Intake among children (4-9 years), adolescents (10-17 years) and adults (18-55 years) by gender

|  | **4-9 y**ears | | | **10-17 y**ears | | | **18-55 y**ears | | |
| --- | --- | --- | --- | --- | --- | --- | --- | --- | --- |
|  | Total | Males | Females | Total | Males | Females | Total | Males | Females |
| **Water** | **51%** | **50%** | **53%** | **54%** | **52%** | **55%** | **54%** | **51%** | **58%** |
| *Bottled water* | *13%* | *13%* | *13%* | *19%* | *19%* | *20%* | *19%* | *20%* | *19%* |
| *Tap water* | *38%* | *36%* | *39%* | *34%* | *34%* | *35%* | *35%* | *31%* | *38%* |
| **Milk & derivatives** | **28%** | **28%** | **28%** | **16%** | **16%** | **17%** | **10%** | **8%** | **11%** |
| **Hot beverages** | **1%** | **1%** | **1%** | **3%** | **3%** | **3%** | **10%** | **11%** | **9%** |
| *Coffee* | *0%* | *0%* | *0%* | *1%* | *1%* | *1%* | *2%* | *1%* | *2%* |
| *Tea* | *1%* | *1%* | *1%* | *2%* | *2%* | *3%* | *8%* | *9%* | *7%* |
| **SSB** | **13%** | **14%** | **12%** | **21%** | **23%** | **20%** | **19%** | **20%** | **17%** |
| *CSD* | *2%* | *3%* | *1%* | *5%* | *6%* | *4%* | *4%* | *5%* | *3%* |
| *Juice-based drinks* | *5%* | *4%* | *5%* | *4%* | *4%* | *5%* | *3%* | *3%* | *3%* |
| *Functional beverages* | *5%* | *5%* | *5%* | *7%* | *8%* | *6%* | *5%* | *6%* | *4%* |
| *RTD tea & coffee* | *2%* | *2%* | *1%* | *5%* | *5%* | *5%* | *6%* | *6%* | *6%* |
| *Flavored water* | *0%* | *0%* | *0%* | *0%* | *1%* | *0%* | *0%* | *0%* | *0%* |
| **100% fruit juices** | **6%** | **7%** | **6%** | **4%** | **3%** | **4%** | **3%** | **2%** | **3%** |
| **A/NSD** | **0%** | **0%** | **0%** | **1%** | **1%** | **0%** | **1%** | **1%** | **0%** |
| **Alcoholic beverages** | **0%** | **0%** | **0%** | **1%** | **2%** | **0%** | **5%** | **7%** | **2%** |
| **Other beverages** | **0%** | **0%** | **0%** | **0%** | **0%** | **0%** | **0%** | **0%** | **0%** |

*SSB* Sugar sweetened beverages, *CSD* Carbonated sweetened beverages, *RTD* Ready to drink, *A/NSD* Artificial/non-nutritive sweetened beverages

**Figure S1** Percentage (%) of participants drinking more or less than the adequate intake (AI) of water from fluids set by European Food Safety Agency (2010) by age group and sex

*M* Males, *F* Females

**Figure S2** Distribution of daily total fluid intake (mL/day), over 7-day of children (4-9 years), adolescents (10-17 years) and adults (18-55 years)

**Figure S3** Percentage of participants (%) meeting the adequate intake (AI) of water from fluids set by the Chinese Nutrition Society according to city socioeconomic status and region.

**Figure S4** Mean daily intake of different fluid types (mL/day) by city socioeconomic status

*SSB* Sugar sweetened beverages, *A/NSB* Artificial/non-nutritive sweetened beverages

**Figure S5** Mean daily intake of different fluid types (mL/day) by region

*SSB* Sugar sweetened beverages, *A/NSB* Artificial/non-nutritive sweetened beverages
